# Well-Loved Music Robustly Relieves Pain: A Randomized, Controlled Trial

**DOI:** 10.1371/journal.pone.0107390

**Published:** 2014-09-11

**Authors:** Christine Hsieh, Jian Kong, Irving Kirsch, Robert R. Edwards, Karin B. Jensen, Ted J. Kaptchuk, Randy L. Gollub

**Affiliations:** 1 Department of Psychiatry, Massachusetts General Hospital and Harvard Medical School, Boston, Massachusetts, United States of America; 2 Program in Placebo Studies and the Therapeutic Encounter (PiPS), Harvard Medical School and Beth Israel Deaconess Medical Center, Boston, Massachusetts, United States of America; 3 Department of Psychology, Plymouth University, Plymouth, United Kingdom; 4 Department of Anesthesiology, Brigham and Women’s Hospital, Boston, Massachusetts, United States of America; 5 Department of Radiology, Athinoula A. Martinos Center for Biomedical Imaging and Massachusetts General Hospital, Charlestown, Massachusetts, United States of America; Cognitive Brain Research Unit, Finland

## Abstract

Music has pain-relieving effects, but its mechanisms remain unclear. We sought to verify previously studied analgesic components and further elucidate the underpinnings of music analgesia. Using a well-characterized conditioning-enhanced placebo model, we examined whether boosting expectations would enhance or interfere with analgesia from strongly preferred music. A two-session experiment was performed with 48 healthy, pain experiment-naïve participants. In a first cohort, 36 were randomized into 3 treatment groups, including music enhanced with positive expectancy, non-musical sound enhanced with positive expectancy, and no expectancy enhancement. A separate replication cohort of 12 participants received only expectancy-enhanced music following the main experiment to verify the results of expectancy-manipulation on music. Primary outcome measures included the change in subjective pain ratings to calibrated experimental noxious heat stimuli, as well as changes in treatment expectations. Without conditioning, expectations were strongly in favor of music compared to non-musical sound. While measured expectations were enhanced by conditioning, this failed to affect either music or sound analgesia significantly. Strongly preferred music on its own was as pain relieving as conditioning-enhanced strongly preferred music, and more analgesic than enhanced sound. Our results demonstrate the pain-relieving power of personal music even over enhanced expectations.

**Trial Information:**

Clinicaltrials.gov NCT01835275.

## Introduction

Music is a treatment that can affect pain perception through a complex set of past and present cues. Juslin and Vastfjall (2008), Salimpoor et al. (2011), and Koelsch (2009), among others, posited many ways through which music may activate regional brain networks mediating reward and anxiolytic effects that also overlap with regions involved in analgesia [1]–[9]. At the same time, music is an enactor of strong perceptual and behavioral responses, such as action tendency and emotional regulation [10], which may also contribute to modulation of pain perception. Scientific reports of pain relief date back to at least the time when Gardner (1960) used it to treat 1000 dental patients and found that using music and white noise, one quarter of the patients did not need additional analgesia during a dental procedure [11]. Amongst more recent music analgesia studies, positive emotions to music were seen to be a modulator of pain [12], but others did not find that music’s cognitive and emotional effects were as influential on pain ratings as treatment expectancy [13]. Specific factors contributing to music analgesia have still not been established definitively [14].

No less complex than music are placebo responses, whose definition has evolved to emphasize psychosocial and contextual cues that generate objectively measurable psychobiological effects [15]. More specifically, beliefs, expectations, and previous treatment are all factors on the patient side that can influence the clinical outcome. Graded doses of increasing amounts of positive clinical contextual factors steadily produce more positive results [16]. Furthermore, research has begun to elucidate the underlying neurochemical and neuroanatomical substrates of placebo responses [3], [17], [18]. Expectancy in particular has emerged as one of the most well supported factors contributing to placebo analgesia [19], [20]. Studies have shown that boosting expectations through conditioning leads to enhanced analgesia, as well as the opposite effect of hyperalgesia with induced negative expectations [21], [22]. This linkage between expectancy, conditioning, and analgesia has been shown across a variety of conditioned stimuli, including acupuncture, creams, and visual cues [18], [23], [24]. Because of the countless ways we may modify subtle attitudes and actions in clinical circumstances, examining factors such as expectancy is of high interest for clinical translation of experimental analgesic outcomes.

To investigate whether the analgesic effect of an emotionally pleasurable stimulus, such as strongly preferred music, could be modified by the well studied conditioning paradigm used within placebo analgesia, we designed an experiment that juxtaposed music with conditioned-expectancy enhancement in two opposing ways - one in which conditioning aimed to enhance expectations of relief for music, and the second in which music would be put up against conditioned expectancy enhancement of a non-musical control sound. We used a well-validated conditioning procedure [19], [25]–[28] of pairing lower heat pain stimuli with a target audio cue to achieve boosted analgesia. Our control group examined the analgesic effects of music and control sound alone. We were able to achieve the aim of shedding light on whether the analgesia of music would be enhanced or disrupted by expectancy-based placebo mechanisms.

## Methods

### Ethics Statement

All study procedures were carried out with Institutional Review Board approval from MIT COUHES (protocol #1206005109) and MGH (protocol #2012P000969), approved on June 27, 2012 and June 31, 2012 respectively. Data was collected at the Martinos Imaging Center in Charlestown, MA. All participants were taken through written informed consent prior to initiating any study procedures. The protocol for this trial and supporting CONSORT checklist are available as Checklist S1 and Protocol S1. This trial was registered under clinicaltrials.gov as trial #NCT01835275. Registration was completed after subject recruitment had begun due to a delay in assigning the appropriate Responsible Party to the record. The authors confirm that all ongoing and related trials for this intervention are registered.

### Participants

Participants met the following criteria: ***Inclusion*** - healthy male and female adults aged 18–50, body mass index <30. ***Exclusion*** - current major medical, neurological, or psychiatric disease, pregnancy, advanced music training, instability of responses to experimental pain (see Study Procedures Section), non-fluent speaker of English, BDI-II (Beck Depression Inventory) [29], [30] score greater than 13, previous experience in pain experiments, current or previous ear/nose/throat or hearing issues compromising ability to listen to audio stimuli. Volunteers were recruited by advertising via email, web, and bulletin board announcements. Recruitment took place from July 1, 2012 to January 23, 2013, and all study procedures were completed within the period from July 2012–January 2013.

### Study Procedures

**Figure 1:**
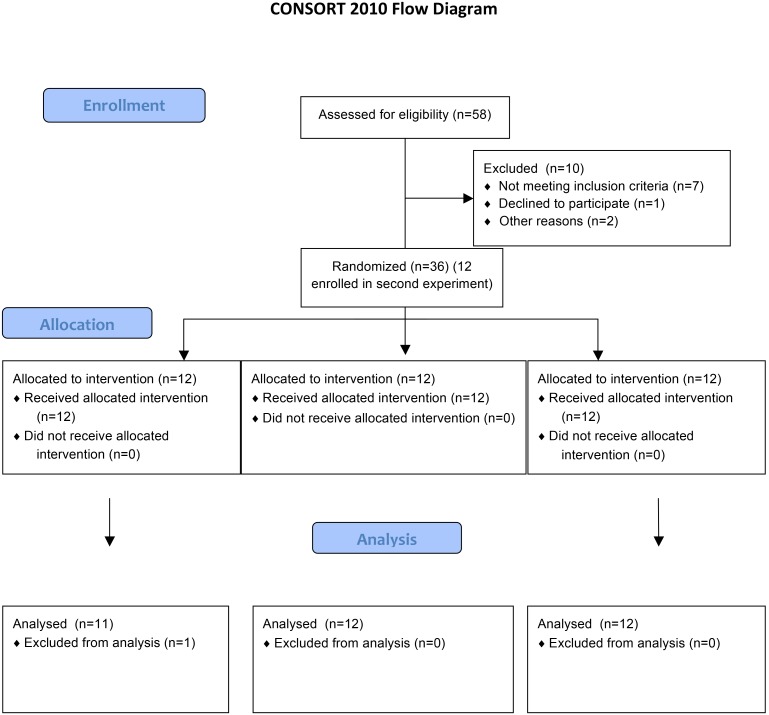
CONSORT Flowchart.

A two-session experiment was performed with 48 healthy, pain experiment-naïve participants. In a first cohort, 36 were randomized (via assignment to outcomes from a random number generator) into 3 treatment groups, including music conditioning, non-musical sound conditioning, and no-conditioning. The primary author completed the random sequence generation, enrollment, and participant assignments. Primary outcome measures included the change in subjective pain ratings to calibrated experimental noxious heat stimuli, as well as changes in treatment expectations on an Expectancy of Relief Scale (ERS). The ERS is a 0–10 scale (with 0 indicating “does not work at all” and 10 indicating “complete pain relief”) used to measure the expectation of treatment pain relief [18]. We also acquired subjective responses to treatments assessed through semi-free form interviews and surveys. A separate replication cohort of 12 participants received only music conditioning following the main experiment. The flow of participants through the study can be found in Figure 1.

The two behavioral sessions were separated by a minimum of 2 days and a maximum of 10 days. Prior to coming to the experimental site, all participants were asked to acquire/choose a set of personal music that satisfied certain criteria for the study (see next section). During both sessions, participants received sets of calibrated noxious thermal stimuli using a Thermal Sensory Analyzer (TSA-II) or the Pathway CHEPS model (Contact Heat-Evoked Potential Stimulator) with a 3 cm×3 cm probe (Medoc Advanced Medical Systems, Rimat Yishai, Israel) running proprietary computerized visual analog scale software (COVAS). Each stimulus was 12 seconds long (2.5 second ramps with 7 second plateaus), and was delivered in blocks of 5 with a jittered average of 30 seconds between stimuli. After each stimulus, participants used 0–100 visual analog scales to rate the pain intensity and unpleasantness, with anchors of 0 = no pain and 100 = worst imaginable pain.

In the *first session*, participants underwent the consent process and screening questions, and we determined whether participants could report consistent, appropriate responses to the calibrated noxious thermal stimuli. Anchors for the pain ratings scales were explained verbally prior to administering the first set of stimuli. In the *second session* we repeated testing for appropriate responses to the thermal stimuli – only participants that performed consistently on the pain rating task (could reliably rate mild intensity pain stimuli as less painful than moderate intensity stimuli and have comparable ratings across sessions - within about 1 STD) would continue to further testing during the second session.

**Figure 2:**
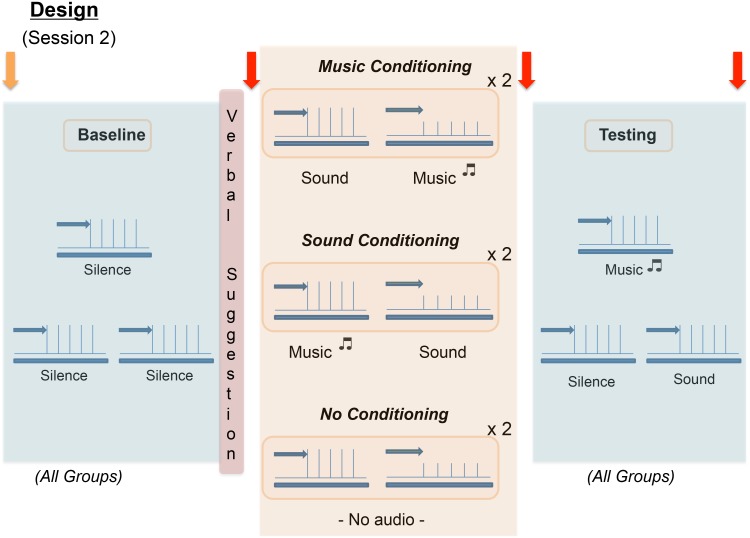
Schematic of Session 2. All subjects first receive identical baseline stimuli on three spots of their right arm. Conditioning groups were then given a verbal suggestion specific to their conditioned audio target, while the no-conditioning group was given a neutral suggestion. All groups then received four blocks of two stimulus intensities, with levels determined during Session 1 (target levels = 20–40 out of 100 and 55–70 out of 100 on the pain intensity VAS). Conditioning groups received lower heat levels when the conditioned audio stimulus was on, while the no-conditioning group received stimuli with silence. All groups were then tested with three audio conditions: music, sound, and silence, in randomized order across participants. Within each set of trials, lead-in arrows represent 1 minute of audio or silence that preceded the start of pain stimuli. In all groups ERS was assessed right after verbal suggestion, then following conditioning/variable pain, and after testing (red arrows). We added one more baseline ERS assessment within the music conditioning replication group at the end of session 1 (orange arrow).

Participants who were eligible to continue first received identical baseline stimuli (calibrated individually during Session 1) on three spots of their right arms, in silence (Figure 2). Participants in conditioning groups then received a conditioning- expectancy manipulation procedure designed to enhance expectations of pain relief in response to their assigned treatment [26]. Participants in conditioning groups were first told that during the intended conditioned stimuli they could experience less pain: “Today we’re testing a treatment for pain that is not yet in clinical use. The reason we’re using ____ (this special frequency-filtered sound, your specially selected music) is because previous studies have shown it to have pain-relieving properties. This is what we’re testing today, and we’re just comparing it to ____ (the sound, the music), and silence.” We then proceeded to pair the target conditioning stimuli with lowered pain levels, with the other audio (music or sound) paired with the baseline pain level. In the no-conditioning control group, we stated that *either* their music or the sound could have analgesic effects. Participants in this group then received two pain levels, with no audio paired. Finally, all groups underwent testing with stimuli identical to baseline, on the same three spots, within audio conditions of music, sound, and silence. All stimuli during the conditioning/variable pain block were applied to regions on the *left* arm, to avoid over-stimulation of regions on the right arm. We randomized order of low and high pain stimuli in the conditioning/variable block, and order of audio presentation during testing, across subjects. This resulted in equal numbers of subjects receiving each possible audio order within each group.

Expectancy was assessed with the ERS at time points including post-verbal suggestion, post-conditioning/variable pain, and post-testing (Figure 2). Expectancy ratings for both music and sound analgesia were requested in the no-conditioning group, as we treated music and sound equally within our verbal suggestion, and to provide baseline measures of ERS as compared to conditioned music and sound. Additionally, each subject rated their mood/valence, arousal, and perceived control using the three scales of the Self Assessment Manikin [31], and answered questions about their experience with the pain and audio after all baseline and testing blocks. At the end of the study all participants completed a final questionnaire assessing their belief in their specific treatment’s efficacy, as well as providing further details on their experience with both their chosen sound and music samples. Questions were framed to neutrally assess opinion for both the effect of the sound and music samples.

In the music conditioning replication cohort that was enrolled after completion of the initial study, we added an explicit reminder that they would be receiving stimuli identical to that from baseline during the conditioning block to better ensure high expectations of music analgesia. The purpose of this group was to verify the effects of expectancy-enhancement on music analgesia in the first music conditioning group. The same ERS measures were taken as in the original cohort, and in addition we obtained one further ERS score at the end of Session 1 (orange arrow in Figure 2) *prior to* administration of the verbal suggestion for analgesia.

### Music and sound sample criteria and choices

To determine individual song choices, we used a list of criteria designed to specify a particular controlled set of participants’ personal music choices for analgesia. Prior to coming to the first study visit, all participants were asked to provide a set of songs (each at least 4 minutes long) that they love, had been familiar with for at least a few years, could listen to repeatedly, varied across a range from very relaxing to very energizing, did not evoke specific memories or chills, and that they had not seen the music video for. We excluded songs associated with specific visual memories including music videos in order to concentrate our study more on strong emotions associated with the music itself [4], [32], and songs that consistently induced chills to ensure a more even set of strong emotional responses from all participants [1]. Participant songs were screened for these criteria during song rating and characterizing in Session 1. We created 4-min. song excerpts centered on the most favored part of each piece and of length appropriate for the pain trial blocks. To give further background on the participants’ musical backgrounds, we excluded those with advanced music training (greater than 3 years of serious voice, instrument, or theory study), while including those with previous common music exposure limited to examples such as “music classes during grade school” or “a couple years of violin class when I was 12”. Music preferences included a variety of genres, such as alternative, to country, to pop. On average, participants regarded music as “pretty important” to them.

The non-musical sound samples consisted of alternating, frequency-filtered noise clips that were chosen based on studies where participants perceived filtered noise to be less distressing than white noise [33], [34]. During Session 2, all participants chose the song they wanted to listen to during the pain trials prior to their first exposure to audio. A list of these songs can be found as Music List S1.

### Analysis

The primary outcomes were pre-post changes in pain intensity and unpleasantness. For the first cohort these data were analyzed using a 3×3 (conditioning by audio type) mixed model analysis of variance (ANOVA) with conditioning (music, sound, or none) as the between-subjects factor, and audio modality (music, sound or silence) as the within-subjects factor. We then compared pain intensity and unpleasantness in the replication cohort with the original music group using a 2×3 (group by audio) mixed model ANOVA. Bonferroni corrections were used for post hoc comparisons following significant main effects.

We examined expectancy in two ways. Participants in the no-conditioning group, where music and sound were presented equally, rated expected relief for both music and sound. These data were analyzed using a 3×2 (time by audio type) repeated measures ANOVA. Participants in the music and sound conditioning groups rated expected relief only for the audio type that was the focus of the verbal suggestion and conditioning. These data were analyzed using a 3×2 (time by conditioning) mixed model ANOVA. Statistical analyses were done using IBM SPSS Statistics version 20. Final sample sizes for sufficient power to detect outcomes were determined during an interim analyses conducted during data collection, based on previous work completed with the current study’s methods by the laboratory. Finally, to analyze the semi-free form qualitative interview and survey data, we performed thematic analyses following the procedure and recommendations of Braun and Clark [35] to extract the themes most relevant to our research questions. All data was first coded, then iteratively collated and refined to maximize internal homogeneity and external heterogeneity of final themes. Extracts in the discussion were selected based on the strength of their congruency with the final themes.

## Results

We enrolled 58 participants, from which 10 were dropped due to the following factors: the requirement of stable and reliable responses to pain stimuli necessary to perform the quantitative experiments, not wishing to continue, or not following study procedure guidelines. We studied 12 participants in each of four groups for a total of 48 participants (32 females), with average age 27±7. 36 of these comprised the initial cohort for which the main experiment was conducted. 12 participants served as a replication sample for the music conditioning group. All participants completing study procedures were included in the final analyses within originally assigned groups.

### Pain Ratings

Examination of the distribution of changes in pain intensity and unpleasantness ratings revealed extreme positive kurtosis for intensity change scores for music (3.99). A box plot revealed one extreme outlier in the music conditioning group. With data from this participant removed, skewness and kurtosis for these six variables were all between +/−1. Hence, subsequent analyses were conducted with data where this one outlier was removed.

**Figure 3:**
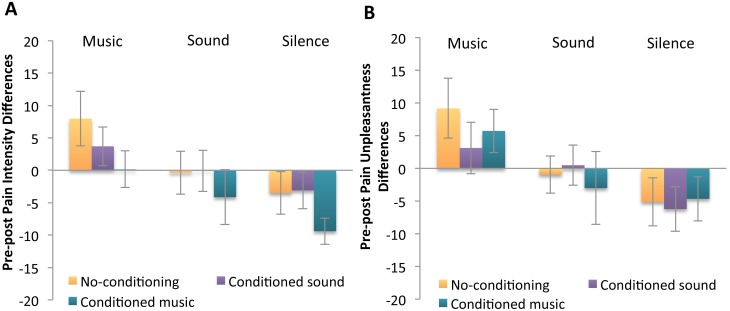
Average pain intensity and unpleasantness changes, all original groups. N = 35. Shown here are pre (baseline) minus post (testing) pain difference scores (mean ± SEM); positive values indicate analgesia. Panel A displays Pain Intensity, panel B displays Pain Unpleasantness.

Table 1 displays baseline (pre) and test block (post) ratings of pain intensity and unpleasantness. Pre-post changes in these ratings are displayed in Figure 3. The 3×3 ANOVA on changes in pain intensity in the primary cohort revealed a significant main effect for audio type, F (2,64) = 15.01, *p*<.001, eta^2^ = .32. Paired contrasts using a Bonferroni adjustment for multiple contrasts revealed that music decreased pain intensity significantly compared to both sound (p = .026) and silence (p<.001), whereas the difference between sound and silence failed to reach significance (p = .059). Neither the main effect for conditioning nor the conditioning by audio type interaction reached significance. It appears that the superiority of music over sound held regardless of conditioning. The 2×3 ANOVA comparing the original music conditioning group to the replication conditioning group showed a similar main effect for audio type, F (2,42) = 7.38, *p* = .002, eta^2^ = .26, with music decreasing pain (*p* = .051) and silence increasing it (*p* = .057).

**Table 1:** Average Pain Ratings for all Groups.

Conditioning	Post Audio	Pain Intensity		Pain Unpleasantness	
		*pre*	*post*	*pre*	*post*
None	Music	55(13)	47(15)	46(20)	37(20)
	Sound	56(16)	56(15)	46(22)	47(20)
	Silence	58(15)	61(14)	48(23)	54(23)
Music	Music	59(19)	59(16)	50(19)	44(16)
	Sound	59(22)	63(16)	48(19)	51(15)
	Silence	57(16)	66(15)	49(16)	54(15)
Sound	Music	50(17)	47(17)	45(23)	42(24)
	Sound	49(14)	49(19)	44(21)	43(23)
	Silence	51(19)	55(20)	44(24)	51(25)
Music replication	Music	55(18)	48(15)	43(24)	35(19)
	Sound	55(15)	55(14)	43(21)	44(22)
	Silence	57(18)	61(14)	44(26)	49(23)

Mean (SD) ratings of pain intensity and unpleasantness for each audio type before and after the conditioning/variable pain phase for both cohorts. Note that all pre- pain stimuli occurred in silence (i.e. pre- ratings for music and sound are given for the corresponding skin spot of stimulation). One outlier subject has been removed from the original music conditioning group (as noted in text).

The 3×3 ANOVA on changes in pain unpleasantness in the primary cohort revealed a significant main effect for audio type, F (2,64) = 15.43, p<.001, eta^2^ = .33. Paired contrasts using a Bonferroni adjustment for multiple contrasts revealed that music decreased pain unpleasantness significantly compared to both sound (p = .015) and silence (p<.001), and the difference between sound and silence just reached significance (p = .05). Neither the main effect for conditioning nor the conditioning by audio type interaction reached significance. For this aspect of the pain experience as well, it also appears that the superiority of music over sound, and of sound over silence at a statistical trend level, held regardless of conditioning. The 2×3 ANOVA comparing the original music conditioning group to the replication conditioning group showed a similar main effect for audio type, F (2,42) = 8.44, *p* = .001, eta^2^ = .29, with music decreasing pain unpleasantness (*p* = .013) and no significant difference between sound and silence increasing it (*p* = .174).

### Expectancy

**Figure 4:**
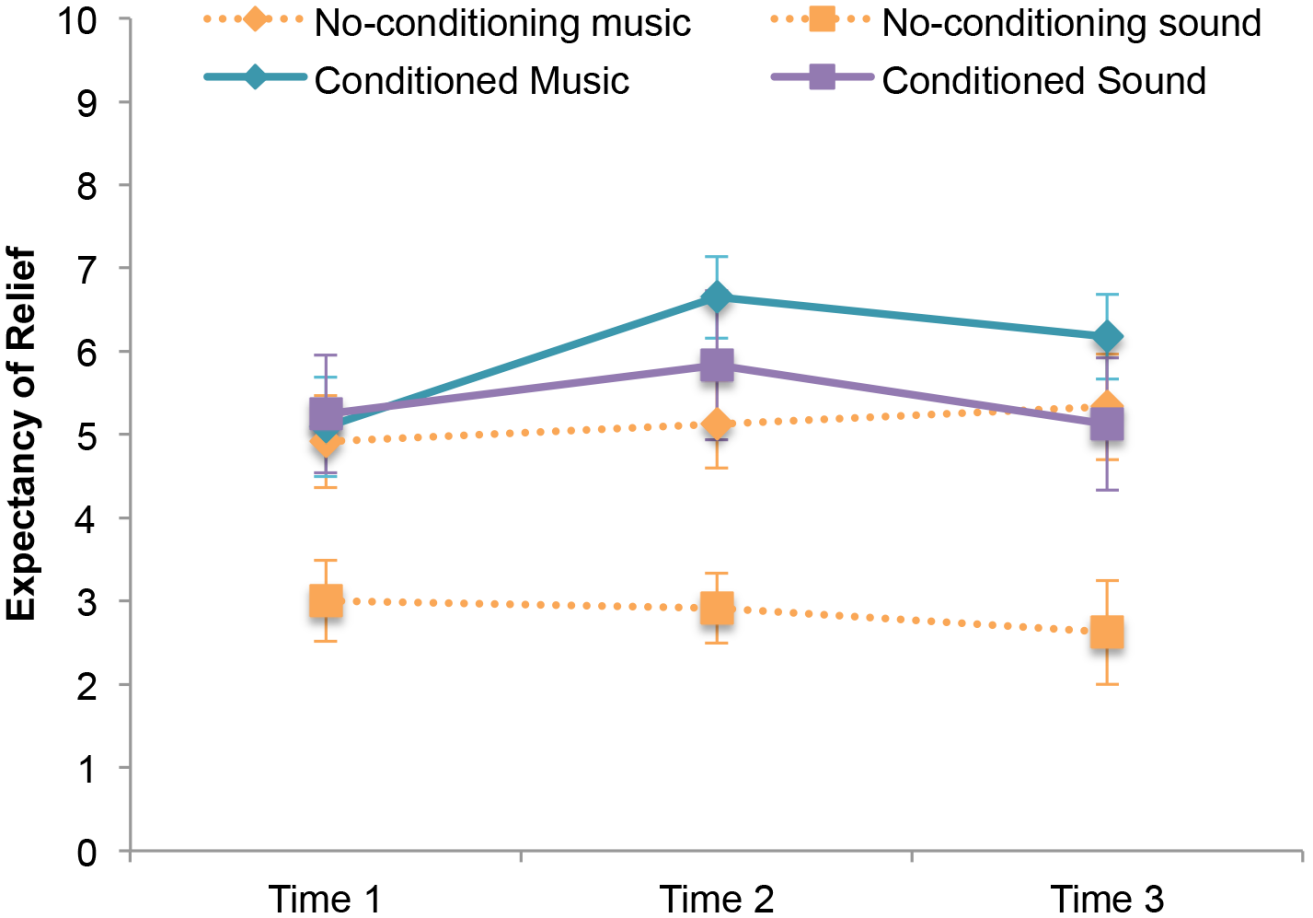
Average expectancy scores in all original groups, across all ERS assessment points . Means ± SEM are shown for N = 35. Time 1 occurs after verbal suggestion but prior to the conditioning phase in the conditioning groups, and prior to the matched variable pain phase in the no- conditioning group. Time 2 occurs immediately after the conditioning/variable pain phase, and Time 3 occurs after testing.

The ANOVA in the no-conditioning group revealed a main effect for audio type, F (1,11) = 43.70, p<.001, eta^2^ = .80). These participants expected much greater pain relief from music (Mean = 5.08; SD = 1.69) than from sound (Mean = 2.89; SD = 1.42), an effect that accounted for 80% of the variance. Neither the main effect for time nor the time by audio type interaction approached significance. The ANOVA in the two conditioning groups revealed a main effect for time, F (2,42) = 6.05, p = .005, eta^2^ = .22. Polynomial contrast revealed a significant quadratic effect, F (1,21) = 13.31, p = 002, eta^2^ = .39, in which expected relief increased following conditioning and then decreased after post-conditioning testing. The main effect for conditioning group did not approach significance. The group by time interaction was not significant (p = .096). Expectancy scores are shown in Figure 4. One participant in the music conditioning group failed to provide a pre-conditioning expectancy rating.

## Discussion

Without conditioned-expectancy enhancement, music had significantly higher measured positive expectancy and was most effective at decreasing pain, followed by sound over silence. Expected relief was boosted in the conditioning groups, however typical corresponding increases in analgesia [26], [36]–[38] were not observed – the significant changes in expectancy did not link to pain relief. Music was the most pain relieving irrespective of conditioning, with enhanced music expectancy adding no further analgesia.

While some routes to analgesia, such as distraction and positive expectations of pain relief, have been seen to be additive [39], our experiment shows that preferred music and conditioning-enhanced expectancy do not appear to be, though our results are tempered by somewhat small group sizes. The powerful relationship individuals had with their music was displayed by the significantly higher expectancy for music in the no-conditioning group that explained 80% of the variance in expectancy ratings. Indeed, these high expectations could be indicative of overlapping mechanisms between music and expectancy-based placebo analgesia that may have precluded an analgesic boost from our conditioning procedure. We saw evidence that musical expectancy [8] and its associations with pleasure were in effect here, as our thematic analysis revealed that subjects regularly identified specific aspects of their songs that would “draw them in” and that they favored. The music selected for use in this experiment could have strongly activated dopaminergic pleasure centers and specifically the nucleus accumbens [5], [9], implicated within analgesia as well [40]. These reward pathways could then have interacted at many levels with the opioidergic system to mediate analgesia [7], [41], [42]. In one preliminary study, results showed that an opiate receptor antagonist could potentially reduce the pleasurable chills response to music [43]; in a later PET study, music-induced chills activated the PAG region, an endogenous source of opiates [1]. Strong evidence exists that placebo analgesia expectations are also a form of reward expectancy [44]–[46] contributing to pain relief through representation of a more favorable homeostatic state. Thus, we would expect music and expectancy-based placebo analgesia to share at least some common pathways. This may explain in part why boosting expectancy did not necessarily enhance analgesia in our music conditioning group.

Additionally, the preferred personal music could have yielded maximal experimentally detectable endogenous analgesia in a ceiling effect. We believe that the strength of the analgesic effects were likely due to the latent conditioned relationships subjects had with their songs, leading to a more complex view of music as an analgesic agent. We suggest it can be viewed in the same framework that placebo responses are increasingly being seen in - that of a rich, nuanced set of contextual personal factors. An individual’s song becomes connected over time to people, places, and circumstances such that there is a robust latent effect from the history and time spent with the music. Indeed, additional analyses of systematically gathered, qualitative participant experiences revealed themes consistent with those seen in the nascent field of neurobehavioral music research [14]: of *deep relationship with their music: “music has gotten me through a lot of hard times…it moves me regardless of mood, pain, emotion”; “I was really into my song. As in, I felt like I was in my song.”; of music absorption*, where specific aspects of their music engaged them strongly, akin to Zatorre and Salimpoor’s [8] concept of rewarding dynamics of musical expectancies. These “nodes” of pleasure could serve as “waypoints”, as one subject described, during the pain experience. Further analyses of our data supported the existence of previously defined analgesic components of music, including perceived control and positive valence [12], [13], [47], [48], and active music usage [49]. Subjects were able to consciously acknowledge bolstered feelings of control and positive valence: *“The specific music I chose makes me feel like I’m going somewhere, and can’t be stopped. It makes me feel less vulnerable…”; “Music inherently makes me happy”; “music made me feel…energized and in control of myself”.* These statements were further confirmed by our qualitative review of perceived control and valence in the Self Assessment Manikin results. With respect to increased control, we saw a theme of a*ctive coping and musical distraction* in that many participants reported purposely focusing on the music or that it was effective as a distraction from the pain: *“…chose this music because it was upbeat and energizing, which I knew would distract me”; “I focused on the song, listening to the lyrics and also knowing when the song, and my pain, was going to end”*. Strikingly, the heat pain was even felt to be the distractor rather than the center of attention for one subject*: “If anything it was more bothersome/unpleasant because it distracted me from the music”.* Consistent with previous efforts to define and isolate specific components of music analgesia, it is becoming clear that these factors are all distinct contributors.

We further propose that enhancing expectations for sound may not have boosted analgesia precisely because the conditioning phase, with lowered pain during sound samples, also included trials with powerfully rewarding music. If conditioning based expectancy modulation works through pathways common to music, then the presence of highly personal music during our attempted generation of increased sound expectancy would certainly interfere with that process. It would be akin to trying to show the glow of a lamp when the sun is shining brilliantly into the room. This cognitive conflict brings to mind the framing of belief and its effects on learning that Corlett et al (2010) put forth: “…sculpted connections…predict subsequent states of the internal and external world and respond adaptively; however, should that next state be surprising, novel or uncertain new learning is required.” [50]. Most likely it was quite surprising for subjects in this group to feel greater pain to music than sound when their previous belief in their music undoubtedly would lead them to expect otherwise. Thus, this kind of prediction error could also have interfered with the manipulation process. Overall the findings indicate that social suggestion and first-hand experience of a phenomenon can be powerful enough to influence conscious expectation, but other factors from music and its long conditioned history with individuals may dominate endogenous mechanisms of pain relief. There appears to be much more behind music analgesia not captured by reports on expectancy scales. Our results show that two media (music and sound) of similarly boosted expectancy can still perform very differently for pain relief.

One caveat to mention is the somatotopic specificity that has been shown for placebo analgesia [28], which may have limited the conditioning effect possible in our paradigm to the left arm (where conditioning was carried out), versus the right testing arm. However, we believe it is unlikely that subjects’ higher expectations post-conditioning would not transfer over to the right arm for the duration of the final three testing spots, as placebo analgesia is mainly mediated through the central nervous system and can thereby have effects in any part of the body. Still, future experiments may examine the conditioning effects when the test skin areas can be closer to the conditioning spots. An additional limitation of this experiment is that we do not know if non-musical sound can be conditioned when music is not present in the conditioning and testing phases; we note also that our small sample size may have precluded enough power for significance in some of our more specific comparisons. However, that sound expectancy was successfully boosted indicates that the procedure was not ineffective. With respect to the conditioning procedure, it is possible that the temperature difference must be even greater to enhance analgesia, though a difference too great can garner suspicion that a manipulation has taken place. Finally, though the interaction between time and group for the expectancy boost did not reach significance, we saw a trend towards a difference between music and sound conditioning. We speculate that with larger groups, future studies may see an additional boost to music analgesia from the conditioning procedure after all.

## Conclusion

On the surface it seems that music should be amenable as a modulation target for the same reasons contextual placebo factors are being discussed for maximal clinical effect. Yet here, the combination of music with an expectancy-mediated conditioning procedure showed that music analgesia is not improved by these methods. On its own it performed just as well as it did following conditioning. Thus, despite the likely existence of many converging mechanisms of endogenous analgesia, music and expectancy-based placebo analgesia were not additive or synergistic in this study.

In her review, Tracey [7] put forth an intriguing consideration for the amelioration of pain: “…closely related to the subjective interpretation of a sensory stimulus is the concept of meaning…Even suffering can be rewarding if it has meaning to the sufferer.” Music is indeed a medium filled with deep personal meaning, following closely alongside most of us as we wind our ways through life. With continued systematic study of healing components, we may still learn how to create whole new combinations of environments and contextual elements that lead to the alleviation of suffering.

## Supporting Information

Checklist S1 **CONSORT checklist for reporting randomized trials.**
(DOC)

Music List S1 **A list of the final song choices (with artist listed) that participants of each group chose to listen to during pain testing blocks in Session 2.**
(DOCX)

Protocol S1 **The full original detailed protocol submitted to the Partners Human Research Committee.**
(DOC)

## References

[1] Blood AJ, Zatorre RJ (2001) Intensely pleasurable responses to music correlate with activity in brain regions implicated in reward and emotion. Proc Natl Acad Sci U S A 98: 11818–11823. 11573015 10.1073/pnas.191355898 PMC58814

[2] Koelsch S, Fritz T, DY VC, Muller K, Friederici AD (2006) Investigating emotion with music: an fMRI study. Hum Brain Mapp 27: 239–250. 16078183 10.1002/hbm.20180 PMC6871371

[3] Benedetti F, Carlino E, Pollo A (2011) How placebos change the patient’s brain. Neuropsychopharmacology 36: 339–354. 20592717 10.1038/npp.2010.81 PMC3055515

[4] Juslin PN, Vastfjall D (2008) Emotional responses to music: the need to consider underlying mechanisms. Behav Brain Sci 31: 559–575 discussion 575–621. 18826699 10.1017/S0140525X08005293

[5] Salimpoor VN, Benovoy M, Larcher K, Dagher A, Zatorre RJ (2011) Anatomically distinct dopamine release during anticipation and experience of peak emotion to music. Nat Neurosci 14: 257–262. 21217764 10.1038/nn.2726

[6] Koelsch S (2009) A neuroscientific perspective on music therapy. Ann N Y Acad Sci 1169: 374–384. 19673812 10.1111/j.1749-6632.2009.04592.x

[7] Leknes S, Tracey I (2008) A common neurobiology for pain and pleasure. Nat Rev Neurosci 9: 314–320. 18354400 10.1038/nrn2333

[8] Zatorre RJ, Salimpoor VN (2013) From perception to pleasure: Music and its neural substrates. Proc Natl Acad Sci U S A 110 Suppl 2 10430–10437. 23754373 10.1073/pnas.1301228110 PMC3690607

[9] Salimpoor VN, van den Bosch I, Kovacevic N, McIntosh AR, Dagher A, et al (2013) Interactions between the nucleus accumbens and auditory cortices predict music reward value. Science 340: 216–219. 23580531 10.1126/science.1231059

[10] Gabrielsson A (2001) Emotions in Strong Experiences with Music. In: Juslin PN, Sloboda JA, editors. Music and Emotion: Theory and Research. New York: Oxford University Press.

[11] Gardner WJ, Licklider JC, Weisz AZ (1960) Suppression of pain by sound. Science 132: 32–33. 13826543 10.1126/science.132.3418.32

[12] Roy M, Peretz I, Rainville P (2008) Emotional valence contributes to music-induced analgesia. Pain 134: 140–147. 17532141 10.1016/j.pain.2007.04.003

[13] Perlini AH, Viita KA (1996) Audioanalgesia in the Control of Experimental Pain. Canadian Journal of Behavioural Science 28: 292–301.

[14] Bernatzky G, Presch M, Anderson M, Panksepp J (2011) Emotional foundations of music as a non-pharmacological pain management tool in modern medicine. Neurosci Biobehav Rev 35: 1989–1999. 21704068 10.1016/j.neubiorev.2011.06.005

[15] Finniss DG, Kaptchuk TJ, Miller F, Benedetti F (2010) Biological, clinical, and ethical advances of placebo effects. Lancet 375: 686–695. 20171404 10.1016/S0140-6736(09)61706-2 PMC2832199

[16] Kaptchuk TJ, Kelley JM, Conboy LA, Davis RB, Kerr CE, et al (2008) Components of placebo effect: randomised controlled trial in patients with irritable bowel syndrome. BMJ 336: 999–1003. 18390493 10.1136/bmj.39524.439618.25 PMC2364862

[17] Hall KT, Lembo AJ, Kirsch I, Ziogas DC, Douaiher J, et al (2012) Catechol-O-methyltransferase val158met polymorphism predicts placebo effect in irritable bowel syndrome. PLoS One 7: e48135. 23110189 10.1371/journal.pone.0048135 PMC3479140

[18] Kong J, Gollub RL, Rosman IS, Webb JM, Vangel MG, et al (2006) Brain activity associated with expectancy-enhanced placebo analgesia as measured by functional magnetic resonance imaging. J Neurosci 26: 381–388. 16407533 10.1523/JNEUROSCI.3556-05.2006 PMC6674420

[19] Montgomery GH, Kirsch I (1997) Classical conditioning and the placebo effect. Pain 72: 107–113. 9272794 10.1016/s0304-3959(97)00016-x

[20] Kirsch I (2000) The response set theory of hypnosis. Am J Clin Hypn 42: 274–292. 10710811 10.1080/00029157.2000.10734362

[21] Kong J, Gollub RL, Polich G, Kirsch I, Laviolette P, et al (2008) A functional magnetic resonance imaging study on the neural mechanisms of hyperalgesic nocebo effect. J Neurosci 28: 13354–13362. 19052227 10.1523/JNEUROSCI.2944-08.2008 PMC2649754

[22] Bingel U, Wanigasekera V, Wiech K, Ni Mhuircheartaigh R, Lee MC, et al (2011) The effect of treatment expectation on drug efficacy: imaging the analgesic benefit of the opioid remifentanil. Sci Transl Med 3: 70ra14. 10.1126/scitranslmed.3001244 21325618

[23] Laverdure-Dupont D, Rainville P, Montplaisir J, Lavigne G (2009) Changes in rapid eye movement sleep associated with placebo-induced expectations and analgesia. J Neurosci 29: 11745–11752. 19776261 10.1523/JNEUROSCI.1224-09.2009 PMC6666668

[24] Kong J, Spaeth R, Cook A, Kirsch I, Claggett B, et al (2013) Are all placebo effects equal? Placebo pills, sham acupuncture, cue conditioning and their association. PLoS One 8: e67485. 23935833 10.1371/journal.pone.0067485 PMC3729687

[25] Voudouris NJ, Peck CL, Coleman G (1989) Conditioned response models of placebo phenomena: further support. Pain 38: 109–116. 2780058 10.1016/0304-3959(89)90080-8

[26] Voudouris NJ, Peck CL, Coleman G (1990) The role of conditioning and verbal expectancy in the placebo response. Pain 43: 121–128. 2277714 10.1016/0304-3959(90)90057-K

[27] Benedetti F, Pollo A, Lopiano L, Lanotte M, Vighetti S, et al (2003) Conscious expectation and unconscious conditioning in analgesic, motor, and hormonal placebo/nocebo responses. J Neurosci 23: 4315–4323. 12764120 10.1523/JNEUROSCI.23-10-04315.2003 PMC6741114

[28] Price DD, Milling LS, Kirsch I, Duff A, Montgomery GH, et al (1999) An analysis of factors that contribute to the magnitude of placebo analgesia in an experimental paradigm. Pain 83: 147–156. 10534585 10.1016/s0304-3959(99)00081-0

[29] Geisser ME, Roth RS, Robinson ME (1997) Assessing depression among persons with chronic pain using the Center for Epidemiological Studies-Depression Scale and the Beck Depression Inventory: a comparative analysis. Clin J Pain 13: 163–170. 9186024 10.1097/00002508-199706000-00011

[30] Turner JA, Romano JM (1984) Self-report screening measures for depression in chronic pain patients. J Clin Psychol 40: 909–913. 6480856 10.1002/1097-4679(198407)40:4<909::aid-jclp2270400407>3.0.co;2-j

[31] Bradley MM, Lang PJ (1994) Measuring emotion: the Self-Assessment Manikin and the Semantic Differential. J Behav Ther Exp Psychiatry 25: 49–59. 7962581 10.1016/0005-7916(94)90063-9

[32] Janata P (2009) The neural architecture of music-evoked autobiographical memories. Cereb Cortex 19: 2579–2594. 19240137 10.1093/cercor/bhp008 PMC2758676

[33] Villarreal EA, Brattico E, Vase L, Ostergaard L, Vuust P (2012) Superior analgesic effect of an active distraction versus pleasant unfamiliar sounds and music: the influence of emotion and cognitive style. PLoS One 7: e29397. 22242169 10.1371/journal.pone.0029397 PMC3252324

[34] Roy M, Lebuis A, Hugueville L, Peretz I, Rainville P (2012) Spinal modulation of nociception by music. Eur J Pain 16: 870–877. 22337476 10.1002/j.1532-2149.2011.00030.x

[35] Braun V, Clarke V (2006) Using thematic analysis in psychology. Qualitative Research in Psychology 3: 77–101.

[36] Kong J, Kaptchuk TJ, Polich G, Kirsch I, Vangel M, et al (2009) Expectancy and treatment interactions: a dissociation between acupuncture analgesia and expectancy evoked placebo analgesia. Neuroimage 45: 940–949. 19159691 10.1016/j.neuroimage.2008.12.025 PMC2737445

[37] Amanzio M, Benedetti F (1999) Neuropharmacological dissection of placebo analgesia: expectation-activated opioid systems versus conditioning-activated specific subsystems. J Neurosci 19: 484–494. 9870976 10.1523/JNEUROSCI.19-01-00484.1999 PMC6782391

[38] Benedetti F, Arduino C, Amanzio M (1999) Somatotopic activation of opioid systems by target-directed expectations of analgesia. J Neurosci 19: 3639–3648. 10212322 10.1523/JNEUROSCI.19-09-03639.1999 PMC6782226

[39] Buhle JT, Stevens BL, Friedman JJ, Wager TD (2012) Distraction and placebo: two separate routes to pain control. Psychol Sci 23: 246–253. 22261568 10.1177/0956797611427919

[40] Baliki MN, Geha PY, Fields HL, Apkarian AV (2010) Predicting value of pain and analgesia: nucleus accumbens response to noxious stimuli changes in the presence of chronic pain. Neuron 66: 149–160. 20399736 10.1016/j.neuron.2010.03.002 PMC2873199

[41] Scott DJ, Stohler CS, Egnatuk CM, Wang H, Koeppe RA, et al (2007) Individual differences in reward responding explain placebo-induced expectations and effects. Neuron 55: 325–336. 17640532 10.1016/j.neuron.2007.06.028

[42] Wager TD, Scott DJ, Zubieta JK (2007) Placebo effects on human mu-opioid activity during pain. Proc Natl Acad Sci U S A 104: 11056–11061. 17578917 10.1073/pnas.0702413104 PMC1894566

[43] Goldstein A (1980) Thrills in response to music and other stimuli. Physiological Psychology 8: 126–129.

[44] de la Fuente-Fernandez R, Ruth TJ, Sossi V, Schulzer M, Calne DB, et al (2001) Expectation and dopamine release: mechanism of the placebo effect in Parkinson’s disease. Science 293: 1164–1166. 11498597 10.1126/science.1060937

[45] Lidstone SC, Schulzer M, Dinelle K, Mak E, Sossi V, et al (2010) Effects of expectation on placebo-induced dopamine release in Parkinson disease. Arch Gen Psychiatry 67: 857–865. 20679593 10.1001/archgenpsychiatry.2010.88

[46] Petrovic P, Dietrich T, Fransson P, Andersson J, Carlsson K, et al (2005) Placebo in emotional processing–induced expectations of anxiety relief activate a generalized modulatory network. Neuron 46: 957–969. 15953423 10.1016/j.neuron.2005.05.023

[47] Mitchell LA, MacDonald RA (2006) An experimental investigation of the effects of preferred and relaxing music listening on pain perception. J Music Ther 43: 295–316. 17348757 10.1093/jmt/43.4.295

[48] Mitchell LA, MacDonald RA, Brodie EE (2006) A comparison of the effects of preferred music, arithmetic and humour on cold pressor pain. Eur J Pain 10: 343–351. 15878297 10.1016/j.ejpain.2005.03.005

[49] Sloboda JA, O’neill SA (2001) Emotions in Everyday Listening to Music. In: Juslin PN, Sloboda JA, editors. Music and Emotion: Theory and Research. New York: Oxford University Press.

[50] Corlett PR, Taylor JR, Wang XJ, Fletcher PC, Krystal JH (2010) Toward a neurobiology of delusions. Prog Neurobiol 92: 345–369. 20558235 10.1016/j.pneurobio.2010.06.007 PMC3676875

