# Supplementary material for: Well-Loved Music Robustly Relieves Pain: A Randomized, Controlled Trial
Source: PLoS One. 2014 Sep 11;9(9):e107390. doi: 10.1371/journal.pone.0107390 (PMC4161415; doi:10.1371/journal.pone.0107390)
Supplement: Music List S1 — A list of the final song choices (with artist listed) that participants of each group chose to listen to during pain testing blocks in Session 2. (DOCX) [file pone.0107390.s002.docx]

Supplementary Material

*Participant song choices during testing*

*(Choices listed as Song, Artist)*

**Music conditioning group**

- Why Georgia, John Mayer
- New Divide, Linkin Park
- How to Save a Life, The Fray
- Ordinary Day, (artist unknown)
- Because I’m Stupid, Kim Hyun Joong
- Wonderwall, Oasis
- Clocks, Coldplay
- Green Light, John Legend and Andre 3000
- Shake it out, Florence and the Machine
- My Love, Justin Timberlake
- Glamorous Indie Rock & Roll, The Killers
- (data for one participant lost)

**New music conditioning group**

- Human Nature, Michael Jackson
- Please don’t stop the Music, Rihanna
- Connected, Stereo MC's
- Night Time, The XX
- Set Fire to the Rain, Adele
- Take what you Take, Lily Allen
- Dig, Incubus
- Lights, Mad Violinist
- The Script, Breakeven
- When I’m Gone, Eminem
- Solsbury Hill, Peter Gabriel
- Dancing, Elisa

**Sound conditioning group**

- Natural High, Bloodstone
- Free as a Bird, The Beatles
- Sweet Child o’ Mine, Aerosmith & Guns N Roses
- How ‘bout us, Champaign
- Only Anarchists are Pretty, The World / Inferno Friendship Society
- Little Lion Man, Mumford and Sons
- Us vs. Them, LCD Soundsystem
- Somebody’s Baby, Jackson Browne
- In the Waiting Line, Zero 7
- If this World Were Mine, Luther Vandross & Cheryl Lynn
- Brown Skin, Erykah Badu
- (data for one participant lost)

**No conditioning group**

- All My Days, Alexi Murdoch
- Just the Two of Us, Will Smith
- Points of Authority/99 Problems/One Step Closer (explicit version), Jay-Z
- Kiss from a Rose, Seal
- Postcards from Italy, Beirut
- Bipolar, Blonde Redhead
- She will be loved, Maroon 5
- Pillow and Records, Aidan Hawkins
- Midnight City, M83
- O.N.E., Yeasayer
- More than a Feeling, Boston
- (data for one participant lost)
