# Supplementary material for: Well-Loved Music Robustly Relieves Pain: A Randomized, Controlled Trial
Source: PLoS One. 2014 Sep 11;9(9):e107390. doi: 10.1371/journal.pone.0107390 (PMC4161415; doi:10.1371/journal.pone.0107390)
Supplement: Protocol S1 — The full original detailed protocol submitted to the Partners Human Research Committee. (DOC) [file pone.0107390.s003.doc]

# PARTNERS HUMAN RESEARCH COMMITTEE

# DETAILED PROTOCOL

PROTOCOL TITLE

Audio-Enhanced Analgesia in Healthy Subjects

**DATE: 04/26/12**

## **I. BACKGROUND AND SIGNIFICANCE**

The experience of pain has both sensory and affective elements, and can be modulated with cognitive distractions as well as emotional manipulation. The neural network involved in analgesia includes both the dopaminergic reward system and a descending pain inhibitory opiodergic system that begins in the cerebral cortex and extends downwards to the spinal cord, with some evidence of additional mediators that antagonize the opiodergic circuit through factors such as anxiety. Placebo analgesia studies have shown an effect from verbal expectancy manipulation, which can be additionally boosted through covert conditioning. More recently, there have been a number of demonstrations of music’s analgesic capabilities in experimental settings^1,2,3,4,5^. Music has been shown to both reduce anxiety and activate reward circuitry^6,7^; because both factors are part of the neural pathways modulating pain perception, it is possible for music to access one or both pathways as an analgesic vehicle.

In this study, we propose to control or measure all previously successful elements of music analgesia. Following the success and feasibility of the personal music choice paradigm in healthy subjects (protocol 2010P002894) and of conditioning (protocols 2011P000748, 2009P000096, 2004P000096), here we implement both for pain experience, which has mind and body components. We intend to harness the potential benefit of familiarity to enhance music analgesia, and will also further the clinical promise of this intervention by using conditioning to augment any effects that music has on pain perception.

**The use of personal music choice in music induced analgesia**

Applied to pain-related disorders, music as a therapeutic modality has a number of advantages over more traditional therapies. Pathologies like depression, often associated with long term illnesses like chronic pain, tend to manifest with different symptoms across cultures^8^, making it difficult to address ethnically and demographically diverse groups with psychotherapy. In the US, the probability of two people of different ethnicities being selected when picked randomly from the country is about 50%. Music has the power to universally be understood: even a culture that has never heard a note of Western music can understand its basic emotionality^9^. It is also becoming highly accessible through services like Pandora Radio and YouTube (both free with Internet access). We know that it can have a strong effect across a variety of people, as even musically untrained (experience only through everyday exposure) listeners can still have strong emotional responses^10^. Perhaps most importantly, music is highly socially accepted, whereas psychotherapy and medication carry much stigma, leading to decreased adherence and possibly even delay or prevented initiation of treatment. Lastly, in contrast to many medications, using music is low risk and has general health benefits^11^, is of lower cost, and is safe, with no drug interactions.

The scientific complexity of personal music choice is great in the context of analgesia. From the effects of choice^4,12^, expectation of relief^12,13^, and potential conditioning from previous exposure^14,15^, to music specific elements including valence/arousal^16,17^, mood dependent preferences, specific memories^18^, and induced chills^19^, the mechanisms operating behind this potentially powerful clinical tool are many. The neural basis of music and emotions is a nascent yet fast growing area of research, and many overlapping regions between pain and music affective circuitry have been found, including limbic, paralimbic, dopaminergic, and hippocampal networks^7^. The history of audioanalgesia itself stretches back to 1959, when Gardner^20^ treated 1000 dental patients and found that using music and noise, ¼ of them did not need any other analgesia during a dental procedure. Through the research proposed in this study, we expect to further increase mechanistic understanding of personal music’s role as a potential analgesic by controlling many of the above factors and testing personal music’s effects in combination with conditioning.

Specifically, we will both verbally induce and measure expectancy of analgesia for their personal music choice. We will also attempt to assess levels of attention and focus on the audio using both scales and interview methods. Balancing carefully between clinical utility and scientific integrity, we are choosing to exclude music choices that invoke specific autobiographical memories and chills, and include a set of individually-selected and characterized songs that are perceived to be highly pleasurable over a range of arousal levels. We will allow individuals to choose their preferred song from that set during the experiment. By requesting that subjects provide songs that cover a range in arousal, we can further explore the uncertainty behind music’s role as a mediator of anxiety or agent of reward^2,3,5,16^,, as higher arousal is generally correlated with anxiety but higher *music*-induced arousal to strongly pleasurable music is correlated with reward. Roy et al (2012) specifically tested arousal to pleasant music for pain modulation, but their samples were not personally chosen by the subjects, and other studies have shown familiarity to mediate reward in the context of arousal^14^. Lastly, conditioning has clearly been able to enhance the analgesic effects of paired stimuli. Because the neurophysiological mechanisms of the effects of music on pain are still largely unclear, the interaction between conditioning and the music element may yield additional insight about the cognitive and affective aspects of music analgesia.

**Experimental manipulations of expectancy**

Positive expectation of pain relief is likely a critical component of context effects in pain treatments, but one that is challenging to tease out from other context effects and manipulate experimentally. Additionally, a problem with studying analgesia using healthy volunteers in experimentally-induced pain studies is that the analgesic effects can be fewer and of lower magnitude than in genuine clinical pain studies^21,22^. This study will take advantage of the fact that positive expectation of treatment related to pain relief can be robustly manipulated in an acute experimental setting using a well-studied conditioning- expectancy paradigm^23-26^. We propose using an experimental manipulation based on the method originally developed by Voudoris and colleagues^23^, who applied a placebo cream as a local analgesic and surreptitiously reduced the pain stimulus. This manipulation successfully produced heightened analgesia to subsequent noxious stimuli. Here, instead of pairing pain reduction with a physical, inert “analgesic” agent, we will pair it with an emotional and cognitive stimulus (music) that has already shown some analgesic effects on its own (see above). As shown before^24^, expectancy and conditioning each have separate importance in the context of analgesia, and we will be able to partially differentiate those effects between our conditioning groups and our non-conditioning group. In all published attempts this conditioning paradigm has been effective in creating robust placebo analgesia sufficient to allow quantitative behavioral measurements; in this study we expect the paradigm to robustly boost whatever analgesic effects the personal music stimulus already has.

**Potential clinical applications**

Though music and conditioning have been studied separately as potential mediators of pain relief, they have not to date been combined in a rigorous, controlled study to test their efficacy together. The studies mentioned thus far have found mostly modest effect sizes, but together these two analgesic methods may be able to more substantially reduce pain. In the case that the effects are not linearly additive, the addition of music into the conditioning-expectancy paradigm links a clinically effective, analgesic perceptual modulation with a modality that is already well integrated into most patient’s lives. Additionally, as music is already a medium that induces associative conditioning of socio-cultural cues, emotions, memories, and identity^7,18^, it may be an ideal candidate for a surreptitiously conditioned analgesic therapy. Piloting of this dual analgesic paves the way to future longitudinal studies of a personal music treatment that is low cost, patient-centered and driven, and can potentially be a lasting, effective relief. This would reduce medication needed and number of medical visits, outcomes that could impact analgesic quantities used post-surgery and by chronic pain patients, and generally decrease the lifestyle impacts that the experience of pain carries with it.

**II. SPECIFIC AIMS**

Music and conditioning to simple cues (e.g light) have each separately shown analgesic effects in experimental pain settings. The goal of this behavioral study is to combine conditioning with both personal music and a control sound sample to test the analgesic effects of these components in healthy subjects.

We expect both conditioning and personal music to have analgesic effects, and that conditioning will further increase the analgesic effects of personal music. To assess the magnitude and specific advantages of music as an analgesic and its potential when paired with conditioning, we will include conditioned chosen sound, non-conditioned chosen sound, and silence as controls in our experiment. The control sound sample serves the important role of controlling for the auditory stimulus modality that music is. In providing a *choice* of sound, our aim is to match the psychological advantages of perceived control, and more strictly test personal music against a relatively innocuous, personally chosen sound.

***Specific Aim****:* This study will combine, separately, specific personal music and chosen sound with conditioning to test whether there are increased conditioning benefits for music vs. sound analgesia on experimental pain in healthy subjects.

***Hypothesis 1****: Highly pleasurable, personally chosen music will reduce pain compared to chosen sound and silence in healthy subjects.*

***Hypothesis 2****: Conditioning will enhance pain reduction when chosen sound or music is on, compared to non-conditioned chosen sound and music.*

***Hypothesis 3****: Conditioning will enhance pain reduction when highly pleasurable, personally chosen music is on, compared to conditioned sound.*

## **III. SUBJECT SELECTION**

In accordance with NIH guidelines, efforts will be made to attain a mix of study participants, in terms of gender and racial/ethnic representation that is reflective of the population of the greater metropolitan Boston area; 63% white, 26% African American and 11% other. Specifically we anticipate that half of our subjects will be male and the other half female and that we will have representation from Asian American, African American and Hispanic minority groups in our final cohorts.

The study will not recruit children under 18 years into this study for several reasons. First there is not sufficient data collected in children documenting the behavioral responses to pain stimuli, or to expectancy manipulations. Second, children may have difficulty understanding and completing the psychometric scales, and struggle with experiencing and rating the pain stimuli.

Inclusion Criteria:

a) Healthy male and female adults aged 18-50

b) Body Mass Index < 30

Exclusion Criteria:

a) Current major medical, neurological, or psychiatric disease

b) Pregnancy

c) Advanced music training

d) Instability of responses to experimental pain (see Study Procedures Section)

e) Non-fluent speaker of English

f) BDI-II (Beck Depression Inventory) score greater than 13

g) Previous experience in pain experiments

h) Current or previous ear/nose/throat or hearing issues compromising ability to listen to audio stimuli

## **IV. SUBJECT RECRUITMENT**

Normal adult volunteers will be recruited by advertising for this study by email, web, and bulletin board announcements posted within/outside the hospital network community according to the protocols approved by the Partners Human Research Committee of Massachusetts General Hospital. All advertisement will be made in accordance with official Internal Review Board policies and constraints. It is not anticipated that additional efforts to recruit minority or female subjects will be required. In addition, the online recruitment system of the MIT Behavioral Research Lab may be utilized as specified in protocol 2010P002894/3. Information to be included in the recruitment notices has been included in this protocol submission.

We will recruit up to 45 subjects or until 36 subjects have completed the study, anticipating an approximately 25% attrition rate for subjects due to inability to meet all continuation requirements (primarily the requirement of stable and reliable responses to pain stimuli necessary to perform the quantitative experiments, as observed in our past and current studies using these methods). All subjects will undergo a telephone or online screening to attempt to distinguish potential subjects from those not meeting eligibility criteria.

During the first visit, one of the study investigators will review all study procedures to be sure subjects are aware of and in agreement with participating in the complete experiment. Written informed consent will be obtained from subjects after all study procedures are described.

Once subjects have met all inclusion criteria, including demonstrated ability to perform the pain rating tasks, they will be randomized into one of three treatment groups – music conditioning, sound conditioning, or routine calibration only.

## **V. STUDY PROCEDURES**

**Overview of Experimental Design**

To accomplish the Specific Aims, a single two-session experiment will be performed with 36 healthy, pain experiment-naïve subjects who will be randomized into 3 treatment groups. The three treatment groups are music conditioning, sound conditioning, and no-treatment routine pain calibration. Primary outcome measures will include the difference (pre- minus post-treatment) in subjective pain rating to calibrated experimental noxious heat stimuli. We will also collect treatment expectations using an Expectancy of Relief Scale (ERS, below) and ratings of subjective responses to treatments assessed through various psychometric scales and surveys for all groups.

The two behavioral sessions will be separated by a minimum of 2 days and a maximum of 10 days. Subjects will be asked to hold common daily activities constant on experiment days (i.e. duration of sleep, eating habits, caffeine intake). Prior to coming to the experimental site, all subjects will be asked to acquire/choose a set of personal music that satisfies certain criteria for the study (see following section). During both sessions, subjects will be receiving sets of calibrated noxious thermal stimuli and after each stimulus will use 0-100 visual analog scales to rate the pain sensation. In the ***first session***, subjects will go through the consent process and screening questions, and we will also determine whether a subject can report consistent, appropriate responses to the application of the calibrated noxious thermal stimuli. This session will last about 120-150 minutes. In the ***second session*** we will repeat testing for appropriate responses to the thermal stimuli – only subjects that perform consistently on the pain rating task (can reliably rate mild intensity pain stimuli as less painful than moderate intensity stimuli and have comparable ratings across sessions - within 1 STD) will continue to further testing during the second session. Subjects who are eligible to continue will be randomized into one of the three treatment groups (N=12/group). These subjects will then receive a modification of a well-characterized conditioning-expectancy manipulation procedure designed to enhance a subject’s expectation of pain relief in response to their assigned treatment. This session will last about 150-180 minutes. At the end of the study all subjects will be de-briefed so that they understand the procedures and why they were used.

**Music and sound sample criteria and choices**

Prior to coming to the experimental site, all subjects will be asked to provide a set of songs (each at least 5.5 minutes long) that they love, have been familiar with for at least a few years but do not tend to get tired of, vary across a range from very relaxing to very energizing, do not evoke specific memories or chills, and that they have not seen the music video for. This list of criteria is designed to maximize our ability to discern the specific effects of subjects’ personal music choices for analgesia. Subjects will be asked to answer a variety of music usage questions prior to starting the study procedures. During Session 1, all subjects will rate their songs in terms of valence and arousal on Self Assessment Manikin (SAM) scales (Bradley and Lang, 1994) and will again be asked if specific memories or chills occur while listening, to screen for these potentially confounding factors^16,18^. Song excerpts centered on the most favored part of each piece and of length appropriate for the intended pain stimulation will be determined. To match the element of choice present in their personal music, subjects will also have the option of choosing one of two sound samples, both neutrally rated in valence and arousal (validated separately by lab members naive to the nature of the control). The sound samples will be colored noise that is frequency filtered to remove higher frequencies and have previously been perceived to be less distressing than white noise^1,5^. Subjects will be exposed to the two sound samples during Session 1 to familiarize them with the stimuli. Then, during Session 2, before any experimental trials begin all subjects will first choose the song and sound sample they want to listen to during the pain trials.

**Session 1: Subject screening, pain response characterization, and training**

After giving consent, all subjects will complete self-report baseline assessments (BDI-II, TAS, STAI-State, GSES, RRQ; see descriptions below). Session 1 is made up of **threshold/tolerance acquisition, screening/calibration**, and **rating stability testing**.

Before beginning heat testing, to ensure that sensitization and habituation of the same area of skin will be avoided in successive stimulation sets, lines will be drawn to divide the skin on the palmar surface of the right forearm into four discrete regions (labeled 1-4 in **Figure 1**). Noxious heat stimuli will be delivered using a Thermal Sensory Analyzer (TSA-II) or the Pathway CHEPS model (Contact Heat-Evoked Potential Stimulator) with a 3 cm ´ 3 cm probe (Medoc Advanced Medical Systems, Rimat Yishai, Israel; Biomedical Engineering Device # 1043681 and #1092912, respectively) running proprietary computerized visual analog scale software (COVAS) (This device is currently used in other studies in our lab, including protocols 2009P000096 and 2010P001368).

The purpose of Session 1 is primarily to train subjects to rate their pain using the 0-100 sensory and unpleasantness visual analog scales (VAS, description below), and to determine individually calibrated stimuli temperatures that elicit certain intensity ratings for each subject. The first block of stimuli will be part of **threshold/tolerance acquisition**, during which subjects will first be instructed on how to use the 0-100 VAS to rate the heat stimuli applied to the skin on the back of their right hand. Each subject will use a button press to start and stop the CHEPS device, running a slowly ascending (.5ºC/sec) series, to indicate both their pain threshold (“as soon as you feel pain”) and their pain tolerance (“as much pain as you can tolerate”). We will run 2-3 series for each of their threshold and tolerance levels, switching to adjacent areas of skin on the back of the hand, for a total of 4-6 series that will yield reliable average values for threshold and tolerance. Then, we will perform up to two ascending series of calibrated heat stimuli on region 1 on the palmar surface of the right forearm (See Figure 1). The first stimulus of each ascending series will be initiated from a resting temperature of 32°C and increased to a target temperature of 38°C. The stimulus will be presented for 12 seconds, including 2.5 seconds each for ramp up and ramp down from resting.


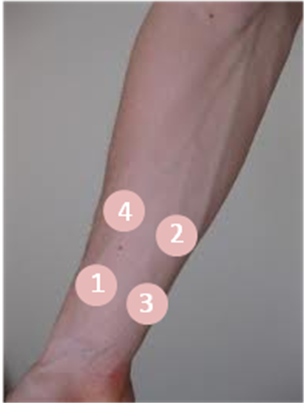
**Figure 1:**

(Right arm)


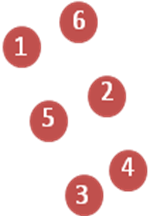
Subsequent stimuli, separated by a minimum of 30 seconds, will be increased by 1°C until reaching 52°C or the subject’s tolerance. Temperatures that elicit subjective intensity ratings of Moderate = 20-40 and Strong = 60-80 will be selected for each subject for use in Session 2. Once the Moderate and Strong heat pain intensities for a subject have been determined, we will initiate **screening/calibration**.

In the **screening/calibration** block, random sequences of Moderate and Strong intensity noxious stimuli will be administered to regions 2 and 3 (See Figure 1). If the subjects can reliably rate the Strong stimuli as more intense than the Moderate stimuli, they will still be eligible to continue.

Finally, during **rating stability testing**, we will administer 5-6 stimuli on region 4 (Figure 1), all at one temperature that elicitsthe subject’s lower Strong intensity ratings to test whether the subject can consistently rate the same noxious heat stimulation. This is the final test during Session 1 for whether subjects can proceed to Session 2.

During Session 1, subjects will also learn to rate their sense of Perceived Control after pain stimuli using the control subscale of the SAM, their valence and arousal to their chosen music using the SAM, their attention to the audio, and their anxiety during pain stimuli. We will also ask them whether any specific memories or autonomic chills responses occurred during their music, and will ask for a different song sample to be used if this is the case as these are music exclusion criteria in this study (as previously stated). Lastly, subjects will be introduced to both sound samples that they may use during Session 2, and asked to not listen to their music samples on their own until the study concludes.

**Session 2: Expectancy manipulation and testing**

Sessions 1 and 2 will be separated by at least two days (but no more than 10 days) to avoid sensitization to repeated application of the noxious stimuli. Before any pain testing begins in Session 2, subjects will complete the STAI-State and BMIS (descriptions below). The rest of Session 2 is made up of **screening/calibration**, **pre-test/baseline, manipulation** and **post-manipulation test**.

The **screening/calibration** block is the same as that completed in Session 1 to determine eligibility to continue participation in the experiment, with this second pass included to thoroughly check for rating consistency (this procedure significantly improves the quality of the data and interpretability of results). The two sets of random stimuli will be applied to regions labeled ‘RS’ in **Figure 2.** If they pass this screening, they will complete a set of **pre-test/baseline** stimuli of the same procedure as the **rating stability testing** from Session 1, though this time in each of regions 1-3 (Figure 2). Ratings for the first set of baseline stimuli during Session 2 must be within 15% of ratings given during rating stability testing during Session 1 to minimize instability of subject rating across sessions. Throughout the administration of noxious pain sequences, subjects will again rate each stimulus using the 0-100 scales immediately after they are presented. After each set of noxious stimuli, subjects will additionally rate their perceived control, valence/arousal, attention, and anxiety.


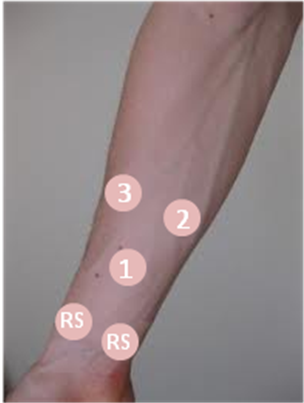


**Figure 2:**

(Right arm)

Before the experimental manipulation, all subjects will choose the music and sound samples they wish to proceed with in the experiment that day. The two emotionally equivalent samples of sound presented in Session 1 will be re-presented for subjects to choose from. Subjects will not listen to their music samples to determine their choice, but rather will visually pick from their list. They will be given about five minutes to complete this task. Subjects will then be randomized to one of three groups (N=12/group), with the groups characterized by conditioning type (music, sound, or none/routine calibration), for the **manipulation** block. After choosing their sound and music samples, subjects in conditioning groups will be told that during the intended conditioned stimuli (see next paragraph) they may experience less pain. In the calibration group, we will state that *either* their music or the sound may have analgesic effects. Subjects will then fill out the ERS indicating the degree to which they anticipate the “treatment” will work as an analgesic.

Within conditioning groups, we will randomly assign subjects to receive either the conditioned stimuli first, or the non-conditioned stimuli. The conditioned stimuli will consist of lower heat stimuli (the subject’s Moderately rated heat level) paired with either music or sound, and non-conditioned stimuli will consist of the same temperature stimuli used in pre-test/baseline paired with the other audio, sound or music. This conditioning procedure will induce a positive expectancy manipulation in addition to the verbal expectancy introduced earlier. In the calibration group, subjects will experience both heat levels, with either the Moderate or Strong pain stimulus first but without any paired auditory stimuli or specific verbal suggestions. Stimuli during the **manipulation** block will be applied to regions 1-4 on the *left* arm, as seen in **Figure 3**, to avoid over-stimulation of regions on the right arm. After each block of stimuli, subjects in conditioning groups will also be asked to indicate whether any specific memories or autonomic chills responses occurred to their music samples, and all subjects may rate their perceived control, valence/arousal to the audio or silence, attention, and anxiety.


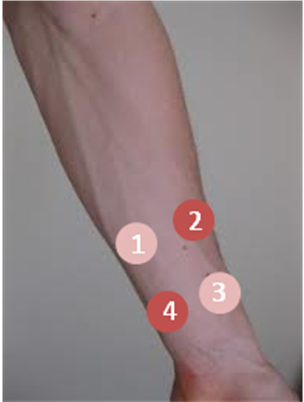


**Figure 3:**

(Left arm; pink is lower heat, red is higher heat)

After **manipulation** they will again fill out the ERS, and then continue to the **post-manipulation test**. This is the same as **pre-test/baseline,** except that stimuli are now presented in three contexts: their chosen music, their chosen sound, and silence, with order of presentation randomized across subjects. Stimuli are again applied to regions 1-3 on the right arm (Figure 2). After each set of audio/silence + noxious stimuli, subjects will additionally rate their perceived control, valence/arousal to the audio or silence, attention, anxiety, and indicate whether any specific memories or autonomic chills responses occurred.

, Subjects will then fill out another ERS indicating their anticipation of their assigned treatment’s analgesic capacity in future treatments, and complete final questionnaires. At this point we may allow subjects to choose either the sound or music sample they listened to previously, and then we will repeat up to two more stimuli blocks with associated ratings identical to those in the post-test. After completion of Session 2 testing procedures, subjects will be debriefed.

**Psychological assessments**

We believe that psychological states may be significant factors in pain experience/modulation and may have an impact on analgesia.

Beck Depression Inventory (BDI): The 21-item BDI has shown good sensitivity and specificity for major depression in chronic pain patients^28,29^. A score greater than 18 will be considered high, and less than 13, low for depression symptoms^28^.

Expectations for Relief Scale (ERS): The ERS is a 0-10 scale (with 0 indicating a very negative expectation of “does not work at all” and 10 indicating a very positive expectation of “complete pain relief”) used to measure the expectation of treatment pain relief^22^.

Tellegen Absorption Scale (TAS): TAS measures individual differences in absorption, a trait that involves an openness to experience emotional and cognitive alterations across a range of situations^30^.  It is a 34-item true-false scales that asks participants to rate the degree to which they become absorbed in everyday imaginative experiences (e.g., viewing a sunset).  It has been widely used in personality research.

Spielberger State-Trait Anxiety Inventory (STAI): STAI consists of two 20-item self-report inventories. It is a rapid but detailed assessment that can distinguish between basal and reactive anxiety. Scores range from 20 to 80 and the higher the score the greater the level of anxiety^31^. Part 1 (trait) measures basal anxiety will be administered during Session 1 and Part 2 (state) which measures reactive anxiety will be administered pre-post-Session 2. Normative data are available^31^.

General Self Efficacy Scale (GSES): This is a 10-item psychometric scale that is designed to assess optimistic self-beliefs to cope with a variety of difficult demands in life. In contrast to other scales that were designed to assess optimism, this one explicitly refers to personal agency, i.e., the belief that one's actions are responsible for successful outcomes^32^.

Rumination Reflection Questionnaire (RRQ): Trapnell and Campbell^33^ originally developed the RRQ to distinguish between the maladaptive and adaptive components of rumination. Rumination items are based on research findings related to metacognitions and evaluations associated with both anxiety and depression. Items on the reflection subscale are from research associated with the cognitive and motivational tendencies typically linked to openness to experience, curiosity, and interest in abstract or philosophical thinking. Each subscale consists of 12 statements to endorse agreement of.

Brief Mood Introspection Scale (BMIS): Consisting of 16 adjectives, two from each of 8 mood categories, it assesses present mood and has been shown to have good factor validity and reliability^34^. We will measure this pre-post-Session 2.

Sensory and Unpleasantness Visual Analog Scale (VAS): This is a simple 0-100 visual scale representing the magnitude of either pain intensity or pain unpleasantness. It has been widely validated and used in studies taking behavioral pain ratings, and instructions are given to subjects on how to distinguish between intensity vs. affective unpleasantness.

Self Assessment Manikin (SAM): A set of three visual scales representing valence, arousal, and perceived control used widely in emotion rating studies.

Final Questionnaire**:** To determine the success of our procedures, we will administer a final questionnaire asking patients whether they believed they had received genuine treatment, how certain they are of the answer, why the treatment might or might not have worked for them, and their final attitudes toward the treatment. We will also ask them various questions about their experience during the procedures.

## **VI. BIOSTATISTICAL ANALYSIS**

**Power Analysis:**
We will study 12 subjects in three groups for a total of 36 subjects; the primary end points are differences between the music/sound conditioning groups and the no conditioning group. In our previous studies for placebo analgesia using a similar model^22, 35-37^, we observed a SD for pain rating difference of 1.7.  Using this value, with n=12 subjects in each group, we expect 80% power to detect an absolute difference in pain ratings of 2.1 or greater (two sample t-test at the 0.05 two-tailed significance level).

**Data analysis**:
The outcome will be the pain rating differences in different conditions. The data will be analyzed using repeated measures analysis of covariance (ANCOVA) with the corresponding control measures (silence) as the covariate, and conditioning status and audio modality (music and sound) as the factor of interest.

## **VII. RISKS AND DISCOMFORTS**

All subjects will undergo repeated assessment of subjective response to brief (~10 sec) noxious thermal stimuli. The application of the heat stimuli may cause minor tissue sensitization. The temperature range used is 36-52 degrees Celsius.

All subjects will complete a set of psychological assessments. Subjects may feel uncomfortable completing some of the questions, and can leave any question blank if they do not wish to answer.

This is a voluntary study in normal adults. The alternative is that the subject does not have to participate. Unwillingness to participate or complete the study will not affect future patient care.

Written informed consent will be obtained after study staff verbally review the consent form with the subject. Subjects will be informed that a physician-investigator will be available at all times to speak with them if they choose.

#### **Protection Against Risks**

All subjects will undergo a telephone or email screening to attempt to distinguish potential subjects from those not meeting eligibility criteria. This procedure will involve answering questions about their medical, psychiatric and pain experience histories. This procedure should take less than 20 minutes for most healthy subjects.

The intensity of pain stimulation will be determined for each subject individually to minimize potential for tissue damage. The maximum temperature to be utilized is below that associated with tissue injury. Subjects will be contacted post-study to document presence or absence of tissue injury at the sites of noxious stimulation. If there is any evidence that the protocol causes more than minor, rapidly reversible sensitization; the protocol will be amended to decrease the frequency and intensity of the noxious stimuli.

During Session 1, subjects will complete a set of psychological assessments. Subjects will be instructed to complete the questionnaires to the best of their ability, but will have the option to leave any question(s) blank. In the unlikely event that evidence of psychological disorder is found (the healthy subjects are screened for psychiatric disorders before entering the study), the principal investigator, Dr. Randy Gollub (a board-certified psychiatrist with 15 years of experience in the Acute Psychiatric Service) will be consulted immediately to direct care as needed.

## **VIII. POTENTIAL BENEFITS**

Subjects will be paid for their participation, but will derive no other direct benefit. The risks to the subjects are minor. The information gained concerning the efficacy of music analgesia and insights into the mechanism underlying the health effects of this form of treatment could have a positive impact on the incorporation of this safe therapeutic treatment modality into medical practice. The risks are well balanced by the potential benefits.

##### Remuneration

Subjects will be paid for their participation. Payments will be as follows:

Subjects will be paid $65 by check at the completion of the study for their participation, and will receive a parking voucher if they request one.

#### **Knowledge to be gained**

It is anticipated that findings from these studies will help advance neuropsychiatric research generally, and knowledge about placebo analgesia, music analgesia, and behavioral mechanisms of pain.

**VIIII. MONITORING AND QUALITY ASSURANCE**

All adverse events will be reported to the Human Research Committee promptly in accordance with guidelines.

All information regarding experimental subjects will be kept in the offices of the Principal Investigator. All data for presentation will be identified by a code number only.

**References**

1. Villarreal EA, Brattico E, Vase L, Østergaard L, Vuust P. [Superior analgesic effect of an active distraction versus pleasant unfamiliar sounds and music: the influence of emotion and cognitive style.](http://www.ncbi.nlm.nih.gov/pubmed/22242169) PLoS One. 2012;7(1). Epub 2012 Jan 5.
2. Knox D, Beveridge S, Mitchell LA, MacDonald RA. [Acoustic analysis and mood classification of pain-relieving music.](http://www.ncbi.nlm.nih.gov/pubmed/21895104) J Acoust Soc Am. 2011 Sep;130(3):1673-82.
3. Roy M, Peretz I, Rainville P. [Emotional valence contributes to music-induced analgesia.](http://www.ncbi.nlm.nih.gov/pubmed/17532141) Pain. 2008 Jan;134(1-2):140-7.
4. Perlini AH, Viita KA. Audioanalgesia in the control of experimental pain. Canadian Journal of Behavioural Science/Revue canadienne des sciences du comportement, Vol 28(4), Oct 1996, 292-301.
5. Roy M, Lebuis A, Hugueville L, Peretz I, Rainville P. [Spinal modulation of nociception by music.](http://www.ncbi.nlm.nih.gov/pubmed/22337476)  Eur J Pain. 2012 Jan 31.
6. Mitchell LA, MacDonald RA. [An experimental investigation of the effects of preferred and relaxing music listening on pain perception.](http://www.ncbi.nlm.nih.gov/pubmed/17348757) J Music Ther. 2006 Winter;43(4):295-316.
7. Koelsch S. Towards a neural basis of music-evoked emotions. Trends in Cognitive Sciences. 1 March 2010. 14(3), 131-137.
8. Gotlib IH and Hammen CL (2009). Handbook of Depression. New York, NY.
9. Fritz T, Jentschke S, Gosselin N, Sammler D, Peretz I, Turner R, et al. (2009). Universal recognition of three basic emotions in music. Current biology : CB, 19(7), 573-6.
10. Bigand, E., & Poulin-Charronnat, B. (2006). Are we "experienced listeners"? A review of the musical capacities that do not depend on formal musical training. Cognition, 100(1), 100-30.
11. Brandes V, Terris DD, Fischer C, Schuessler MN, Ottowitz G, Titscher G, Fischer JE, and Thayer JF. Music Programs Designed to Remedy Burnout Symptoms Show Significant Effects after Five Weeks. Volume 1169, Issue The Neurosciences and Music III Disorders and Plasticity, 422-425.
12. Rose JP, Geers AL, Rasinski HM, Fowler SL. [Choice and placebo expectation effects in the context of pain analgesia.](http://www.ncbi.nlm.nih.gov/pubmed/21850515) J Behav Med. 2011 Aug 18.
13. Kong, J., et al., Brain activity associated with expectancy-enhanced placebo analgesia as measured by functional magnetic resonance imaging. J Neurosci, 2006. 26(2): p. 381-8.
14. Pereira CS, Teixeira J, Figueiredo P, Xavier J, Castro SL, Brattico E. [Music and emotions in the brain: familiarity matters.](http://www.ncbi.nlm.nih.gov/pubmed/22110619) PLoS One. 2011;6(11):e27241.
15. Szpunar KK, Schellenberg EG, Pliner P. [Liking and memory for musical stimuli as a function of exposure.](http://www.ncbi.nlm.nih.gov/pubmed/14979811) J Exp Psychol Learn Mem Cogn. 2004 Mar;30(2):370-81.
16. Salimpoor, V. N., Benovoy, M., Longo, G., Cooperstock, J. R., & Zatorre, R. J. (2009). The rewarding aspects of music listening are related to degree of emotional arousal. PloS one, 4(10)
17. Roy M, Peretz I, Rainville P. [Emotional valence contributes to music-induced analgesia.](http://www.ncbi.nlm.nih.gov/pubmed/17532141) Pain. 2008 Jan;134(1-2):140-7.
18. Janata P. [The neural architecture of music-evoked autobiographical memories.](http://www.ncbi.nlm.nih.gov/pubmed/19240137) Cereb Cortex. 2009 Nov;19(11):2579-94. Erratum in: Cereb Cortex. 2010 Jan;20(1):254-5
19. Blood A and Zatorre RJ (2001). Intensely pleasurable responses to music correlate with activity in brain regions implicated in reward and emotion. Proc. Natl. Acad. Sci. USA 98 : 11818–11823.
20. Gardner, W., Licklider, J. &  Weisz, A. (1960). Suppression of pain by sound, Science, 132, 32.
21. Watson, A., et al., Placebo conditioning and placebo analgesia modulate a common brain network during pain anticipation and perception. Pain, 2009. 145(1-2): p. 24-30.
22. Kong, J., et al., Brain activity associated with expectancy-enhanced placebo analgesia as measured by functional magnetic resonance imaging. J Neurosci, 2006. 26(2): p. 381-8.
23. Voudouris, N.J., C.L. Peck, and G. Coleman, The role of conditioning and verbal expectancy in the placebo response. Pain, 1990. 43(1): p. 121-128.
24. Montgomery, G.H. and I. Kirsch, Classical conditioning and the placebo effect. Pain, 1997. 72: p. 107-113.
25. Price, D.D. and J. Barber, An analysis of factors that contribute to the efficacy of hypnotic analgesia. J Abnorm Psychol, 1987. 96(1): p. 46-51.
26. De Pascalis, V., C. Chiaradia, and E. Carotenuto, The contribution of suggestibility and expectation to placebo analgesia phenomenon in an experimental setting. Pain, 2002. 96(3): p. 393-402.
27. Price, D.D., et al., An analysis of factors that contribute to the magnitude of placebo analgesia in an experimental paradigm. Pain, 1999. 83: p. 147-156.
28. Geisser, M.E., R.S. Roth, and M.E. Robinson, Assessing Depression Among Persons with Chronic Pain Using the Center for Epidemiological Studies Depression Scale and the Beck Depression Inventory: A Comparative Analysis. Clin J Pain, 1997. 13: p. 163-170.
29. Turner, J.A. and J.M. Romano, Self-Report Screening Measures for Depression in Chronic Pain Patients. J Clin Psychol, 1984. 40: p. 909-913.
30. Tellegen, A. and G. Atkinson, Openness to absorbing and self-altering experiences ("absorption"), a trait related to hypnotic susceptibility. Journal of Abnormal Psychology, 1974. 83: p. 268-277.
31. Spielberger, C.D., Gorsuch, R.L., & Lushene, R.E. (1970). Manual for the State-Trait Anxiety Inventory. Palo Alto, CA: Consulting Psychology Press.
32. Schwarzer R, Jerusalem M. Generalized Self-Efficacy scale. Windsor, England.: NFER-NELSON, 1995.
33. Private self-consciousness and the five-factor model of personality: Distinguishing rumination from reflection. Trapnell, Paul D.; Campbell, Jennifer D. Journal of Personality and Social Psychology, Vol 76(2), Feb 1999, 284-304
34. Mayer, J.D., & Gaschke, Y.N. (1988). The experience and meta-experience of mood. Journal of Personality and Social Psychology, 55, 102-111.
35. Kong J, Gollub RL, Polich G, Kirsch I, Laviolette P, Vangel M, Rosen B, Kaptchuk TJ. A functional magnetic resonance imaging study on the neural mechanisms of hyperalgesic nocebo effect. Journal of Neuroscience 2008; 28: 13354-13362. PMID: 19052227 PMCID: PMC2649754.
36. Kong J, Kaptchuk TJ, Polich G, Kirsch I, Vangel M, Zyloney C, Rosen B, Gollub R: Expectancy and treatment interactions. A dissociation between acupuncture analgesia and expectancy evoked placebo analgesia. Neuroimage 2008; 45: 940-9. Epub 2008 Dec 29. PMID: 19159691
37. Kong J, Kaptchuk TJ, Polich G, Kirsch I, Vangel M, Zyloney C, Rosen B, Gollub RL: An fMRI study on the interaction and dissociation between expectation of pain relief and acupuncture treatment. Neuroimage 2009; 47: 1066-76. Epub 2009 Jun 6. PMID: 19501656
